# Global Epidemiology, Health Outcomes, and Treatment Options for Patients With Type 2 Diabetes and Kidney Failure

**DOI:** 10.3389/fcdhc.2021.731574

**Published:** 2021-08-23

**Authors:** Jessica Phillips, Jenny H. C. Chen, Esther Ooi, Janelle Prunster, Wai H. Lim

**Affiliations:** ^1^ Department of Renal Medicine, Sir Charles Gairdner Hospital, Perth, WA, Australia; ^2^ School of Medicine, University of Wollongong, Wollongong, NSW, Australia; ^3^ Depatment of Nephrology, Wollongong Hospital, Wollongong, NSW, Australia; ^4^ School of Biomedical Sciences, University of Western Australia, Perth, WA, Australia; ^5^ Department of Renal Medicine, Cairns Hospital, Cairns, QLD, Australia; ^6^ Medical School, University of Western Australia, Perth, WA, Australia

**Keywords:** diabetes, type 2 diabetes, kidney failure, dialysis, kidney transplant, mortality, post-transplant diabetes, oral hypoglycemic agents

## Abstract

The burden of type 2 diabetes and related complications has steadily increased over the last few decades and is one of the foremost global public health threats in the 21st century. Diabetes is one of the leading causes of chronic kidney disease and kidney failure and is an important contributor to the cardiovascular morbidity and mortality in this population. In addition, up to one in three patients who have received kidney transplants develop post-transplant diabetes, but the management of this common complication continues to pose a significant challenge for clinicians. In this review, we will describe the global prevalence and temporal trend of kidney failure attributed to diabetes mellitus in both developing and developed countries. We will examine the survival differences between treated kidney failure patients with and without type 2 diabetes, focusing on the survival differences in those on maintenance dialysis or have received kidney transplants. With the increased availability of novel hypoglycemic agents, we will address the potential impacts of these novel agents in patients with diabetes and kidney failure and in those who have developed post-transplant diabetes.

## Introduction

The number of people with diabetes has more than doubled over the last two decades, and diabetes has become one of the predominant global health threats of the 21st century. It is estimated that more than 50 million people worldwide have diabetes, and diabetes-related complications and disability are associated with substantial economic healthcare cost and loss of productivity (1).

Type 2 diabetes is the leading cause of kidney failure globally. In Australia, the proportion of treated kidney failure patients with diabetes increased from 42% to 52% between 2005 and 2019, respectively, with diabetes as the primary cause of kidney failure in 39% of patients receiving kidney replacement therapy in 2019 (2, 3). Similar proportions have been reported in the United States (44% in 2012 and 48% in 2017), United Kingdom (20% in 2005 and 30% in 2018), and New Zealand (46% in 2005 and 59% in 2019) (2–7). The increasing prevalence of diabetes in many countries has further contributed to the expanding burden of kidney failure patients, heralding the development of an epidemic of diabetes-related complications worldwide (8).

Similar to the general population (9–11), the presence of type 2 diabetes in patients with kidney failure on maintenance dialysis or received kidney transplants was associated with over a twofold greater risk of cardiovascular disease (CVD) and all-cause mortality compared to patients without diabetes and kidney failure, reinforcing the negative health consequences of diabetes across the health spectrum (12, 13). Given the disproportionate increased risk of CVD and the reduced projected survival in patients with type 2 diabetes and kidney failure, understanding the short- and long-term health risks in this complex patient group, as well as the potential utility of novel anti-diabetic agents are essential when assessing and planning dialysis or kidney transplantation. Furthermore, up to 30% of patients develop post-transplant diabetes mellitus (PTDM) after kidney transplantation, but the diagnosis, risk factors, and the risk of adverse long-term health outcome of this common complication remain inadequately defined. There is considerable uncertainty as to whether the targets of PTDM and treatment options, particularly the availability of novel anti-diabetic agents, should be extrapolated from the general population, and clinicians will need to be cautious of the caveats of inferring findings from the general population to patients with PTDM where the pathophysiology of the disease process, potential drug–drug interactions, and competing comorbidities are vastly different.

This review focuses on the current understanding of the epidemiology and risk of adverse health outcomes of patients with type 2 diabetes and kidney failure and those who have developed PTDM, including the uncertainty in the management strategy and use of novel anti-diabetic agents in these patients.

## Global Burden of Diabetic Kidney Disease: Definition and Incidence

The designation of diabetic kidney disease is to describe the development of chronic kidney disease and kidney failure attributable to the effects of diabetes. The pathogenesis of this disease process is generally conceptualized as the product of prolonged exposure to the toxic effects of hyperglycemia, but it is likely that this process is representative of several comparable and competing pathogenic processes (for example, concurrent hypertensive kidney damage), with the eventual consequence of progressive kidney function decline and ensuing kidney failure (14). The precise estimates of the incidence of chronic kidney disease or kidney failure attributed to diabetes is often underestimated because the diagnosis of diabetic nephropathy was previously established on clinical rather than histological findings. At present, the term diabetic nephropathy is often advocated only in patients with the characteristic lesions of diabetic glomerulopathy established on histology, and diabetic kidney disease occurs in patients with diabetes mellitus and reduced kidney function that can be from many diverse causes, including hypertensive nephrosclerosis and unresolved acute kidney failure (15–17). The biopsy rates to establish the presence of diabetic nephropathy as cause of chronic kidney disease or kidney failure is generally less than 15%, and the variation in the reported incidences of diabetic nephropathy is likely attributed to dissimilarities in clinical practice guidelines and healthcare resources between countries, lack of accurate biopsy data collection, and the heterogeneity of kidney biopsy indications in patients with diabetes (18–20). Misclassification bias of the underlying cause of chronic kidney disease or kidney failure in patients with diabetes is therefore possible, as several population cohort studies have shown that an alternative diagnosis such as glomerulonephritis or a mixed process (evidence of diabetic nephropathy with a second diagnosis) may be relatively common in patients with diabetes (12, 21). Given the availability of novel therapies for diabetes and glomerulonephritis, there is a renewed interest from clinicians and researchers to pursue kidney biopsy more aggressively in patients with diabetes and kidney disease to avoid undue delay recognition and subsequent treatment of the underlying disease. At present, the decision to undertake a kidney biopsy is often determined by the treating clinicians. Even though there is generally no agreement on the criteria for kidney biopsy in patients with diabetes, the presence of “atypical” clinical characteristics such as worsening of proteinuria, presence of active urine sediment, presence of systemic diseases, and absence of concurrent microvascular complications (such as retinopathy) may influence the decision for kidney biopsy (22–25). In addition, kidney biopsy is often useful to establish the presence of non-diabetic disease and quantify the extent of chronic damage in patients with diabetes who have experienced rapid and sustained drop in kidney function or unexplained kidney failure (26, 27). Future research aiming to investigate specific biomarkers (e.g., urine proteomic profile) or distinct clinical and patient phenotypes may assist clinicians to reliably identify patients likely to have diabetic kidney disease and those patients where kidney biopsy should be considered (28, 29).

**Figure 1:**
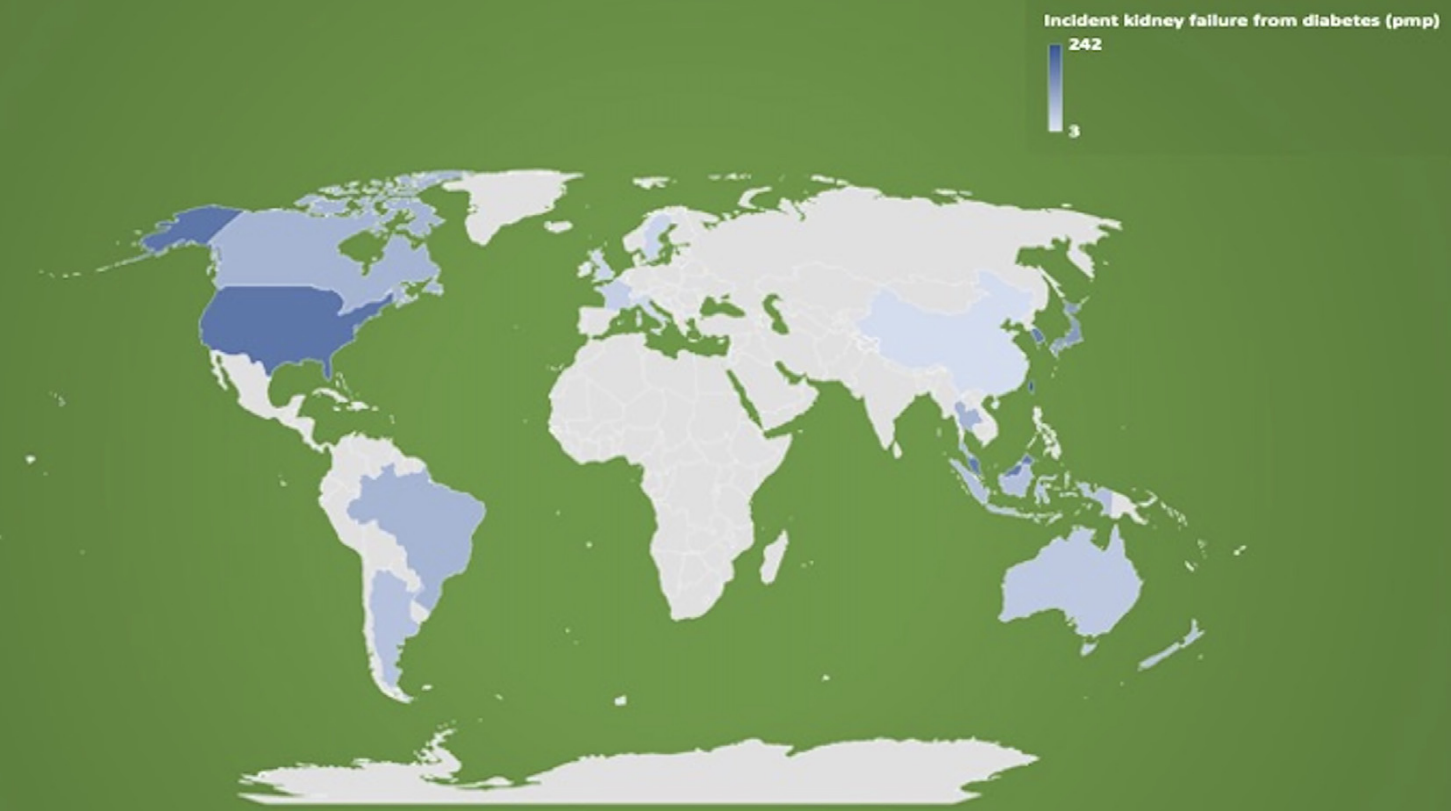
Incidence rates of treated kidney failure patients attributed to diabetes as primary cause of kidney failure (expressed as per million population [pmp] in 2018), by selected countries. Data extracted from the 2020 United States Renal Data System (USRDS) Annual Data Report and the Australia and New Zealand Dialysis and Transplant (ANZDATA) Registry 2019 Report (references 21, 22).

The global incidence of treated kidney failure from diabetic kidney disease varies widely between countries, with the incidence ranging from as low as 10% reported in Romania up to almost 70% in Singapore and Malaysia (30). **Figure 1** shows the incidence rate of patients with treated kidney failure secondary to diabetes by selected countries (2018 data) (31, 32). Although these differences may reflect the true disparities in the population rates of diabetes mellitus and diabetic kidney disease, it is likely that variations in the accuracy and completeness of data captured and the lack of diagnostic criteria for diabetic nephropathy or diabetic kidney disease contributed to these findings. With a greater understanding and potential accessibility and acceptance of conservative, non-dialysis pathway as a treatment option for kidney failure in many countries, the true total incidence of patients with chronic kidney disease or kidney failure from diabetic kidney disease is uncertain but is likely to be considerably underestimated as these patients are often not adequately captured by registries (30). For low- and low-middle-income countries such as Mexico where an estimated 90% of patients with kidney failure do not receive renal replacement therapy and data capture is unreliable, the incidences of untreated and treated kidney failure patients with diabetic kidney disease in these countries are essentially unknown (8). With the temporal change in the survival and pattern of cause-specific deaths in low- and middle-income countries, the burden of kidney failure has substantially increased in parallel with the high-income countries (33). As a way of illustration, several lower-middle- and upper-middle-income Southeast Asian nations have reported substantial increases in the incidence of kidney failure secondary to diabetic kidney disease, with Thailand and the Philippines reporting a respective 1,448% and 378% increase between 2001 and 2015. Other data indicate that this is not an isolated event, with yearly increases in the incidence of treated kidney failure due to diabetic kidney disease documented in many countries surveyed between 2003 and 2016 (33).

## Patients on Dialysis With Type 2 Diabetes: Impact on Long-Term Outcomes

Population cohort studies have consistently shown that the presence of type 2 diabetes is associated with an excess risk of mortality in patients with chronic kidney disease and kidney failure compared to those without diabetes (12, 34–36). However, the association between diabetes status and mortality in kidney failure patients may be modified by several patient and disease characteristics. With the increasing recognition that diabetic nephropathy as the primary cause of kidney failure may represent a distinct clinical and prognostic phenotype compared to patients with type 2 diabetes and kidney failure attributed to non-diabetic nephropathy, re-classification of diabetic nephropathy according to the exact cause has been proposed (37, 38). In a study of 15,419 dialysis patients using data from the European Renal Association-European Dialysis and Transplant Association (ERA-EDTA) Registry, all-cause mortality was 20% higher in patients with diabetes as cause of kidney failure compared to patients with diabetes as a comorbid condition, independent of age and gender (39). These findings were corroborated in a contemporary study from Australia and New Zealand of 56,552 incident dialysis patients between 1980 and 2014, of which 15,829 (28%) had type 2 diabetes and diabetic nephropathy and 4993 (9%) had type 2 diabetes and non-diabetic nephropathy (12). In this study, patients with type 2 diabetes and kidney failure secondary to both diabetic nephropathy and non-diabetic nephropathy have higher risks of all-cause and CVD mortality in the competing risk analysis. Among patients with type 2 diabetes, all-cause (adjusted subdistributional hazard ratio 1.17, 95% confidence intervals 1.10–1.22) and CVD mortality (adjusted subdistributional hazard ratio 1.20, 95% confidence intervals 1.12–1.28) were significantly greater for patients with diabetic nephropathy than for those with non-diabetic nephropathy, emphasizing that these two disease processes may be clinically distinct. Similar to the observation in the general population (40), age modified the association between diabetes and mortality such that the magnitude of the risk of all-cause and CVD mortality was greater in younger patients, particularly those with diabetic nephropathy as cause of kidney failure (12). It is likely that kidney failure attributed to diabetic nephropathy is a surrogate metric of more severe and prolonged duration of diabetes, which may potentially explain the higher risk of mortality observed in this population. However, the possibility of misclassification bias cannot be disregarded as less than 15% of patients with diabetes have a diagnostic biopsy to confirm the underlying cause of kidney failure (12).

The prevalence of newly diagnosed diabetes following dialysis initiation varies between 4% and 16%, with the prevalence rate generally higher in patients maintained on peritoneal dialysis (41–45). However, the difference in the prevalence of newly diagnosed diabetes between peritoneal and hemodialysis patients remains inconsistent (46, 47). Similar to prevalent diabetes, the development of newly diagnosed diabetes is associated with higher mortality risk, although the magnitude of the survival disadvantage is less than those with pre-dialysis diabetes (44, 48).

## Kidney Transplantation in Patients With Type 2 Diabetes: Impact on Long-Term Outcomes

There is often uncertainty with regard to the suitability of kidney transplantation in patients with diabetes and kidney failure, balancing between the expected improved health outcomes from transplantation versus the reduced life expectancy of this population compared to patients without diabetes. Nevertheless, clinicians must be cognizant of the projected incremental gains in survival and improved quality of life following successful kidney transplantation compared with maintenance dialysis treatment, with modeled data showing a substantial survival benefit for patients with diabetes.

In the United States Renal Data System study of 252,358 patients with treated kidney failure under the age of 70 years, kidney transplantation reduced the risk of mortality by over 70% compared to remaining on the waiting list (annual mortality rate of the cohort of 11%). The projected survival gain after transplantation was over 11 years among patients with diabetes, considerably higher than the survival gain in other kidney failure patients without diabetes (49). Similarly, modeled data from Australia and New Zealand showed that wait-listed and transplanted patients with diabetes aged 45 years and 60 years will achieve cumulative incremental survival gains of 1.5 and 0.5 life years, respectively. These modeled scenarios appeared to be cost-effective, with respective incremental cost-effectiveness ratio (ICER) of $8,965 and $21,506 per life years saved, and below the cost-effectiveness threshold of $50,000 for every life year saved (50, 51). These findings suggest that kidney transplantation should continue to be offered for appropriate patients with diabetes and kidney failure.

Despite the survival advantage from kidney transplantation in patients with type 2 diabetes and kidney failure, the long-term survival post-kidney transplantation remains inferior compared to kidney transplant recipients without diabetes. However, recent data from the United States showed a temporal improvement in the survival of kidney transplant recipients with diabetes over the last decade, achieving mortality rates comparable to recipients without diabetes in the most recent era (52). In this study of 1,688 kidney transplant recipients, 413 (24%) had pre-transplant diabetes, of which 75% had type 2 diabetes. The mortality of recipients with diabetes had declined by 12% between 1996 and 2007, which was not observed in those without diabetes. Consequently, the magnitude of the survival disadvantage experienced by recipients with diabetes reduced over time such that after 2004, there was no longer a statistically significant difference in 5-year mortality between kidney transplant recipients with and without diabetes [hazard ratio (HR) 1.45 (95% confidence intervals (95% CI) 0.74, 2.87; *p* = 0.28)] (52). These findings parallel the findings in the general population and suggest that improved management strategies combined with the availability of novel treatment options may have substantially reduced the survival disadvantage of patients with diabetes (53, 54). However, a more contemporaneous study from Australia and New Zealand challenged this initial observation. In this study of 10,714 kidney transplant recipients (9% with type 2 diabetes) spanning almost two decades between 1994 and 2012, the authors reported a significant survival disadvantage of recipients with pre-transplant type 2 diabetes compared to those without diabetes, with the magnitude of this difference more apparent in younger recipients (age <40 years: adjusted HR [95% CI] 5.16 [2.84, 9.35]; age 40–55 years: 2.08 [1.62, 2.66]; >55 years: 1.41 [1.17, 1.71]; referent: no diabetes). There was no temporal improvement in the survival disadvantage of recipients with type 2 diabetes such that the survival difference remains constant across the study time period. CVD and infection were the two main drivers of the higher mortality rates, with recipients with diabetes experiencing almost a threefold increased risk of these complications compared to those without diabetes (13). The disparity in the study findings may reflect the size of the population cohorts (single centre vs. bi-national registry study), inclusion of recipients with and without type 1 and 2 diabetes, and the potential for type II statistical error with a short follow-up time in the study from the United States. Nevertheless, these studies do highlight the continuing survival disadvantage of recipients with diabetes but do raise important questions about the uncertainty in the optimal pre- and post-transplant management strategy of these recipients and whether younger recipients with type 2 diabetes represent a distinct clinical phenotype that are associated with poorer long-term health outcomes. These findings should challenge clinicians and researchers to gain a better understanding of the pathophysiology and natural progression post-transplant of recipients with diabetes and how to integrate novel treatments into the management strategy for kidney transplant candidates and recipients with diabetes.

## Post-Transplant Diabetes Mellitus

### Incidence

PTDM is an established and frequent complication after kidney transplantation, occurring in between 10% and 50% of recipients in the first 12 months post-transplantation (55–60), with an annual incidence of approximately 6%–15% thereafter (47, 61). The observed large variations in the incidence of PTDM are likely related to the frequency and types of screening practices and diagnostic criteria to identify recipients with PTDM, as well as systematic differences in the management of hyperglycemia in the post-transplant period. Consistent with the reported natural history of abnormal glucose regulation in the general population (62), the occurrence of glucose dysregulation is a dynamic process after kidney transplantation, likely related to the progressive reduction in overall immunosuppression including corticosteroids. Several studies have shown substantial movements of kidney transplant recipients between states of normal glucose regulation, pre-diabetes and PTDM beyond 12-months post-transplant. Although a number of recipients with pre-diabetes will progress towards PTDM, there is generally a decline in the proportion of recipients with pre-diabetes and PTDM with up to 1 in 2 recipients with pre-diabetes or PTDM normalizing their glucose regulation on follow-up testing (63–65). Clinicians should be aware of these fluxes between pre-diabetes or PTDM and normal glucose regulation states and should consider re-screening and adapting the management strategy according to the dynamic glucose regulation status, therefore avoiding the unnecessary continuation of oral hypoglycemia treatment(s) or “misclassifying” recipients as having pre-diabetes or PTDM.

### Screening for PTDM

The optimal screening method for PTDM remains unknown, with the Kidney Disease Improving Global Outcomes (KDIGO) 2009 guideline advocating fasting blood glucose, oral glucose tolerance test (OGTT), and/or glycated hemoglobin (HBA1c; level 1C evidence) but the frequency of screening remains unknown (level 2D evidence) (66). However, the screening and formal diagnosis of PTDM can be made from 6 weeks post-transplantation, thereby avoiding the incorrect classification of recipients with transient, postoperative hyperglycemia of little clinical significance. According to the British Clinical Diabetologist and Renal Association guidelines, OGTT is considered the current gold standard for the diagnosis of PTDM (grade 1B evidence: strong recommendation, moderate-quality evidence) (67). Even though the standard diagnostic criteria for diabetes described by the American Diabetes Association (ADA) are utilized (68), transplant clinicians will need to be cognizant of the limitations of when adapting these measures for kidney transplant recipients where a distinct diurnal variation of afternoon and evening hyperglycemia typically occurs (69). The diagnostic utility of a number of screening tests to identify kidney transplant recipients with pre-diabetes and PTDM remains unclear, with unknown thresholds of fasting blood glucose, OGTT, and HBA1c that would provide the best balance between sensitivity and specificity. To use a diagnostic test effectively in clinical practice, clinicians will need to recognize how well each diagnostic test (with the varying thresholds) distinguishes between those recipients with and without pre-diabetes and PTDM (70). However, cohort studies of diagnostic tests to identify kidney transplant recipients with and without pre-diabetes and PTDM are frequently methodologically imperfect, and the reported findings are often not generalizable to cohorts of differing characteristics and clinical practices. Utilization of a single threshold to inform practice may potentially misinform or misclassify recipients with and without pre-diabetes or PTDM (71). The diagnostic utility of other screening tests such as fructosamine, capillary blood glucose, and homeostasis model assessment-insulin resistance (HOMA-IR) score or the combination of a panel of screening tests may provide greater accuracy in identifying those with pre-diabetes or PTDM but will need to be validated in large external population cohorts (60, 72–74). The clinical utility of continuous glucose monitoring post-kidney transplant to identify PTDM or as a means to monitor response to treatment remains unknown.

### Risk Factors

The pathogenesis of PTDM is likely to be multifactorial, with traditional (shared risk factors for type 2 diabetes in the general population) and transplant-related risk factors contributing to this high risk (**Table 1**). While many of the risk factors are non-modifiable, it does help to identify those patients who may benefit from closer monitoring and/or lifestyle intervention post-transplant. The chronic exposure to immunosuppressive agents represents a unique risk factor to kidney transplant recipients, attributed to the toxic or inhibitory (possibly reversible) effects of these drugs to pancreatic islet beta cells and manifesting as insulin resistance or impaired insulin secretion (75–80). There is likely a gradation variation between the different types of immunosuppressive agents and risk of PTDM, with tacrolimus consistently associated with a higher risk of PTDM compared to other agents (55, 81, 82). The associations between several chronic viral infections such as cytomegalovirus (CMV) and hepatitis C with increased risk of PTDM have been observed. The mechanistic pathway for this association remains unclear but may be related to the direct viral-induced toxic effect on beta cells, induction of insulin resistance secondary to hepatic steatosis, and/or excess production of pro-inflammatory cytokines (83–89). Other less recognized risk factors for PTDM include the presence of autosomal dominant polycystic kidney disease and pre- and post-transplant hypomagnesemia, but these associations remain inconsistent and may be related to the residual effects of post-transplant confounding factors (90–95). The failure of magnesium supplementation to improve insulin resistance and glucose metabolism in two small, randomized trials in kidney transplant recipients has challenged the potential causal relationship between hypomagnesemia and the development of PTDM (96, 97).

**Table 1:** Risk factors associated with the development of post-transplant diabetes mellitus.

Risk factors	Potential intervention
**Pre-transplant**	
Recipient age	Non-modifiable, careful monitoring + lifestyle modification post-transplant
Race	Non-modifiable, careful monitoring + lifestyle modification post-transplant
Family history of diabetes	Non-modifiable, careful monitoring + lifestyle modification post-transplant
ADPKD	Non-modifiable, careful monitoring + lifestyle modification post-transplant
HCV infection	Non-modifiable (ensure treatment of HCV), careful monitoring + lifestyle modification post-transplant
Genetic variations	Non-modifiable, careful monitoring + lifestyle modification post-transplant
Obesity	Modifiable, weight loss pre-transplant (± surgical intervention), lifestyle modification post-transplant
**Hypomagnesemia**	Potentially modifiable (with supplementation) + careful monitoring
**Post-transplant**	
Weight gain/obesity	Modifiable, lifestyle modification post-transplant
Hypomagnesemia	Potentially modifiable (with supplementation)
Immunosuppression	
Corticosteroids	Can consider minimization, avoidance, or split-dosing (according to immunological risk)
Calcineurin-inhibitor	Can consider switching from tacrolimus to alternative agents (according to immunological risk)
mTOR inhibitor	Can consider switching to alternative agents (according to immunological risk)
CMV infection	Modifiable, ensure adequate treatment of infection

mTOR, mammalian target of rapamycin; CMV, cytomegalovirus; HCV, hepatitis C virus; ADPKD, autosomal dominant polycystic kidney disease.

### Impact of PTDM on Long-Term Health Outcomes

The association between abnormal glucose regulation and adverse long-term health outcomes in kidney transplant recipients is well established, with an incremental risk of allograft loss, all-cause mortality, fatal and non-fatal CVD, and/or reduced quality of life being apparent from the pre-diabetes stage to the onset of PTDM (56, 98–104). Furthermore, PTDM also contributes to the accelerated atherosclerotic vascular disease burden, particularly in transplant recipients with prevalent vascular disease burden (105, 106). As patients with PTDM share common metabolic CVD risk factors with those with pre-transplant diabetes, the mechanistic pathways of abnormalities in insulin sensitivity and insulin secretion resulting in adverse CVD outcomes are likely to be similar (107–111). A recent population cohort study from Canada showed that kidney transplant recipients with PTDM were 40% less likely to experience major adverse cardiovascular events (MACE) post-transplant but had exhibited similarly high rates of all-cause and CVD mortality compared to those with pre-transplant diabetes (112). In this study, the incidence rates (95% CI) of CVD and all-cause mortality between 1 and 3 years post-transplant for recipients with PTDM were 6.6 (2.5–17.6) and 31.4 (20.5–48.2) per 1000 person-years, respectively. These compared with respective 7.1 (4.7-10.7) and 25.9 (21.2, 31.7) per 1000 person-years for recipients with pre-transplant diabetes, suggesting the importance of early screening and identification of recipients with PTDM (112). Predictably, the cost associated with each new case of PTDM is in excess of USD$21,000 by 2-years post-transplant (47), likely related to the diagnosis and treatment of common diabetes-related complications typically observed in the general population including hospitalizations for severe hyper- and hypoglycemia, ophthalmic complications, neurological complications, CVD, and peripheral vascular disease (113–115).

## Treatment

### Prevention of Kidney Failure and Treatment of Diabetes in Patients With Diabetic Kidney Disease

**Figure 2:**
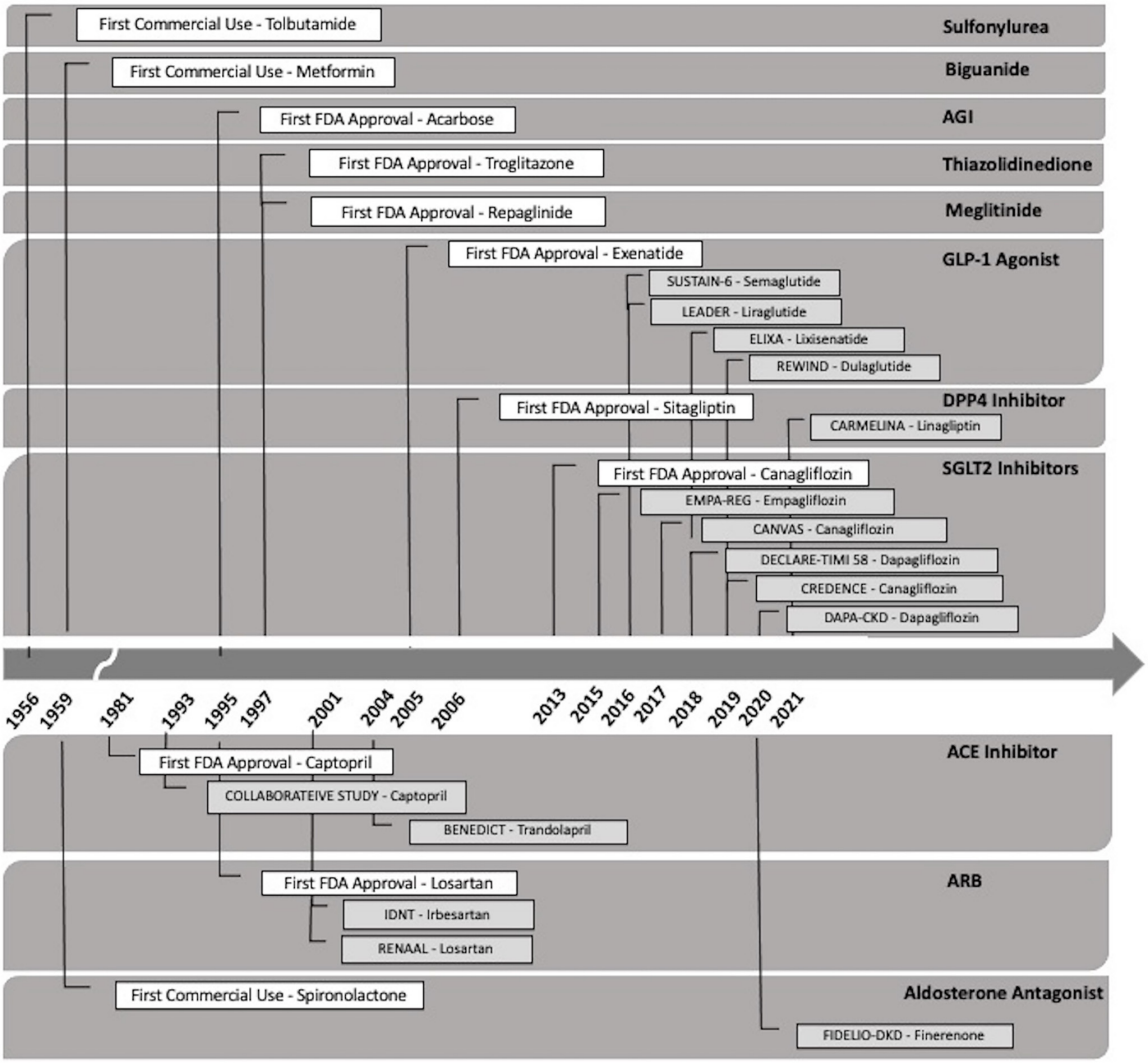
Timeline of the Food and Drug Administration (FDA) approval of the types of oral therapeutic options for the treatment of diabetic kidney disease. DPP4-inhibitor (dipeptidyl peptidase-4 inhibitor), GLP-1 agonist (glucagon-like peptide 1 agonist), SGLT2 inhibitor (sodium-glucose transport protein 2 inhibitor), AGI (alpha glucosidase inhibitor), ACE-inhibitor (angiotensin-converting enzyme inhibitor), and angiotensin II receptor blocker (ARB).

The landscape of diabetic kidney disease treatment has changed significantly over the past decade, particularly with the emergent evidence of the cardiac and nephroprotective benefits of sodium-glucose cotransporter-2 (SGLT2) inhibitors. The availability of SGLT2 inhibitors and other novel agents may potentially slow the upward trend of the incidence of diabetic kidney disease and improvement in diabetes-related complications (**Tables 2**, **3**). **Figure 2** outlines the timeline of treatment for diabetic kidney disease and clinical trials suggesting positive nephroprotective outcomes (116, 119, 172–184).

**Table 2:** Types and efficacy of therapeutic options in the treatment of diabetic kidney disease.

Treatment of diabetic kidney disease	Name of medication	Glycemic effect (% of HBA1c reduction)*	Dose adjustment in chronic kidney disease^#^	Effect on diabetic kidney disease
**Hypoglycemic Treatment**				
**Insulin**	–	1–2.5	eGFR <30 ml/min/1.73 m^2^: reduce dose	*Neutral*
**Biguanide**	Metformin	1–2	eGFR 30–45 ml/min/1.73 m^2^: reduce dose	*Neutral*
			eGFR <30 ml/min/1.73 m^2^: avoid use	
**Sulfonylureas**	Gliclazide	1–2	eGFR <15 ml/min/1.73 m^2^: avoid use	*Neutral*
	Glimepiride		eGFR <15 ml/min/1.73 m^2^: avoid use	
	Glipizide		eGFR <60 ml/min/1.73 m^2^: initiate with caution	
			eGFR <15 ml/min/1.73 m^2^: avoid use	
	Glyburide		Avoid use	
**Meglitinides**	Repaglinide	1–2 0.6–1.2	eGFR <30 ml/min/1.73 m^2^: initiate with caution	*Neutral*
	Nateglinide		eGFR <30 ml/min/1.73 m^2^: initiate with caution	
**Thiazolidinedione**	Rosiglitazone	0.5–1.4	No dose adjustment	*Neutral*
	Pioglitazone			
**Alpha glucosidase inhibitor**	Acarbose	0.5–0.8	eGFR <30 ml/min/1.73 m^2^: avoid use	*Neutral*
	Miglitol			
**DPP4-inhibitor**	Alogliptin	0.5–0.8	eGFR <45 ml/min/1.73 m^2^: reduce dose	*CARMELINA* (*n* = 6,979) (116) Substantial loss of kidney function ≥50%, kidney failure, or death due to kidney disease: HR 0.98 (0.82–1.18), regression to normoalbuminuria: 1.20 (1.07–1.34), reduction of uACR ≥50%: 1.15 (1.07–1.25).
	Linagliptin		No dose adjustment	
	Saxagliptin		eGFR <45 ml/min/1.73 m^2^: reduce dose	
	Sitagliptin		eGFR <45 ml/min/1.73 m^2^: reduce dose	
	Vildagliptin		eGFR <60 ml/min/1.73 m^2^: reduce dose	
			Not to use in combination with GLP-1 agonist	
**GLP-1 agonist**	Dulaglutide	0.5–1.5	eGFR <30 ml/min/1.73 m^2^: avoid use of Exedine-4-based agents (lixisenatide, exenatide)	*Meta-Analysis* (ELIXA, LEADER, SUSTAIN-6, EXSCEL, REWIND; *n* = 56,006) (117)
	Exenatide			
	Liraglutide			
	Lixisenatide		eGFR <15 ml/min/1.73 m^2^: initiate with caution for human GLP-1 based agents (dulaglutide, liraglutide, semaglutide)	Macroalbuminuria, substantial loss of kidney function, kidney failure, or death due to kidney disease: HR 0.83 (95% CI 0.78–0.89), mainly macroalbuminuria
	Semaglutide			
			Not to use in combination with DPP4-inhibitor	
**SGLT2 inhibitor**	Canagliflozin Dapagliflozin Empagliflozin	0.6–1.2	eGFR <30 ml/min/1.73 m^2^: avoid initiation	*Meta-analysis* (EMPA-REG, CANVAS, DECLARE-TIMI 58, CREDENCE; *n* = 38,723) (118) Dialysis initiation, transplantation, or death due to kidney disease: RR 0.67 (95% CI 0.52–0.86), development of kidney failure: RR 0.65 (0.53–0.81), substantial loss of kidney function, kidney failure, or death due to kidney disease: RR 0.58 (0.51–0.66)
				*DAPA-CKD* (*n* = 4,304) (119)
				Substantial loss of kidney function, kidney failure, or death due to kidney or cardiovascular disease: HR 0.61 (0.51–0.72), substantial loss of kidney function, kidney failure, or death due to kidney disease: HR 0.56 (0.45–0.68).
**Treatment for Diabetic Kidney Disease**				
**ACE-inhibitor**	Benazepril	–	eGFR <30 ml/min/1.73 m^2^: reduced dose (except fosinopril: no dose adjustment; perindopril: not recommended.)	*Meta-Analysis* (*n* = 6,819) (120)
	Captopril			Doubling of serum creatinine: RR 0.68 (95% CI 0.47–1.00), kidney failure: 0.60 (0.39–0.93), macroalbuminuria: 0.45 (0.29–0.69)
	Enalapril			
	Fosinopril			
	Lisinopril		>30% increase in creatinine: reduced dose	
	Perindopril		Hyperkalemia: reduced dose	
	Quinapril			
	Ramipril			
	Trandolapril			
**ARB**	Azilsartan	–	No dose adjustment	*Meta-Analysis* (*n* = 3,251) (120) Doubling of serum creatinine: RR 0.79 (95% CI 0.67–0.93), kidney failure: 0.78 (0.67–0.91), macroalbuminuria: 0.49 (0.32–0.75)
	Candesartan			
	Irbesartan		>30% increase in creatinine: reduced dose	
	Losartan		Hyperkalemia: reduced dose	
	Olmesartan			
	Telmisartan			
	Valsartan			
**Aldosterone antagonist**	Spironolactone *Eplerenone*	–	eGFR <30 ml/min/1.73 m^2^: avoid use	*Meta-Analysis* (*n* = 1,243) (121) Effect on uACR −10.9 mg/mmol (95% CI −26.2–4.32), eGFR −3.2 mml/min/1.73 m^2^ (−5.4 to −0.95), CrCl −2.5 ml/min (−7.1–2.0)
	*Finerenone*		eGFR<25 ml/min/1.73 m^2^: limited data	*FIDELIO – DKD* (*n* = 5,734) (122) Substantial loss of kidney function ≥40%, kidney failure, or death due to kidney disease: HR 0.82 (95% CI 0.73–0.93).
**Endothelin receptor antagonists**	Atrasentan	–	eGFR<25 ml/min/1.73 m^2^: limited data	*SONAR* (*n* = 2,648) (123) Doubling of serum creatinine, kidney failure, or death due to kidney disease: HR 0.65 (95% CI 0.49–0.88), doubling of serum creatinine: 0.61 (0.43–0.87), kidney failure: 0.73 (0.53–1.01), 50% eGFR reduction: 0.73 (0.55–0.98)
**Protein kinase C-ß inhibitor**	Ruboxistaurin	–	Limited data	*Study B7A-MC-MBBR* (*n* = 707) (124) No significant difference was observed for uACR or eGFR.
**Selective Janus kinase 1 and 2 inhibitor**	Baricitinib	–	eGFR <25 ml/min/1.73 m^2^: limited data	Phase 2 Trial (*n* = 129) (125) Albuminuria: least squares mean difference 0.59 (95% CI 0.38–0.93). No significant difference was observed for eGFR or serum creatinine.
**Anti-inflammatory, antiproliferative, and antifibrotic agent**	Pentoxifylline	–	eGFR <15 ml/min/1.73 m^2^: limited data	PREDIAN (*n* = 169) (126) Mean difference of eGFR at 24 months: 4.3 ml/min/1.73 m^2^ (95% CI 3.1–5.5), mean difference in percentage increase in urine albumin excretion at 24 months: 20.6% (28.3%–12.9%)

DPP4-inhibitor, dipeptidyl peptidase-4 inhibitor; GLP-1, glucagon-like peptide 1; SGLT2, sodium-glucose transport protein 2; ACE-inhibitor, angiotensin-converting enzyme inhibitor; Angiotensin II Receptor Blockers, ARB; HBA1c, glycated hemoglobin; eGFR, estimated glomerular filtration rate; CKD, chronic kidney disease; uACR, urine albumin/creatinine ratio; HR, hazard ratio; RR, relative risk; CI, confidence interval.

*Accuracy declines in patients with advanced chronic kidney disease or kidney failure.

^#^Dose adjustment recommendations vary between countries.

**Table 3:** Mechanism of actions and clinical concerns of potential diabetes treatment options in kidney failure patients maintained on dialysis or have received kidney transplants.

Drug	Mechanism of action	Increase insulin sensitivity (or reduce insulin resistance)	Increase insulin secretion	Benefits	Risks	Dialysis	Kidney transplant
**Insulin (** 127–134 **)**	Upregulates GLUT4 translocation and uptake of glucose into cells	–	–	Rapid glycemic control	Weight gain, hypoglycemia		
**Biguanides (** 135–141 **)**	Uncertain mechanism of action. Reduces hepatic gluconeogenesis and increases peripheral glucose uptake	Yes **✔**	No **✖**	Potential weight loss, low risk of hypoglycemia	GI intolerance, B12 deficiency, lactic acidosis		
**Sulfonylureas (** 142–146 **)**	Bind ATP-sensitive potassium channels on pancreatic beta cells, stimulating the release of insulin	No **✖**	Yes **✔**	Rapid glycemic control	Weight gain, hypoglycemia		
**Thiazolidinediones (** 147–153 **)**	Binds PPAR-γ on adipocytes, affecting fatty acid metabolism. Reduced hepatic gluconeogenesis. Increases peripheral insulin sensitivity	Yes **✔**	No **✖**	Low risk of hypoglycemia	Weight gain, fluid retention, heart failure		
**DPP4-inhibitors (** 154–161 **)**	Blocks DPP4 enzymatic breakdown of incretin hormones, including GLP-1. Stimulates insulin secretion. Inhibits glucagon secretion	No **✖**	Yes **✔**	Low risk of hypoglycemia	Joint pain, pancreatitis		
**GLP-1 agonist (** 162–167 **)**	Synthetic analogues of GLP-1. Increases insulin secretion, inhibits glucagon secretion. Slows stomach emptying, reduces appetite	No **✖**	Yes **✔**	Cardiovascular benefit. Weight loss. Low risk of hypoglycemia	GI intolerance		
**SGLT2 inhibitors (** 9, 168–171 **)**	Inhibits sodium-glucose co-transporter 2 in the proximal tubule, increasing glycosuria	Possibly	No **✖**	Cardiovascular benefit. Weight loss. Low risk of hypoglycemia. Slows progression of kidney disease	Volume depletion, genitourinary infections, ketoacidosis		

Color legends: ◼ denotes likely to be safe to use, ◼ denotes possibly safe to use, ◼ denotes use is contraindicated. DPP4-inhibitor, dipeptidyl peptidase-4 inhibito; GLP-1, glucagon-like peptide 1; SGLT2, sodium-glucose transport protein 2; eGFR, estimated glomerular filtration rate; GI, gastrointestinal; PPAR-γ, peroxisome proliferator-activated receptor gamma; GLUT-4, glucose transporter type 4; ATP, adenosine triphosphate.

### Diabetes Screening in Patients With Kidney Failure

The optimal HBA1c target associated with a reduction in the risk of hard clinical outcomes in kidney failure patients remains unclear. A systematic review published in 2017 showed a lack of superiority of more intensive (HbA1c < 7%) compared to liberal glycemic control (HbA1c > 7%) for the outcomes of kidney failure, death, or cardiovascular complications, whereas the Action to Control Cardiovascular Risk in Diabetes (ACCORD) post-hoc analysis showed that intensive glycemic control in high-risk patients with type 2 diabetes and mild/moderate chronic kidney disease was significantly associated with over 30% increased risk of cardiovascular and all-cause mortality (185, 186). These findings suggest that the extrapolation of general population data for HBA1c targets may not be appropriate for patients with kidney failure. Consequently, both the KDIGO and ADA guidelines have therefore advocated individualized glycemic target for patients with advanced chronic kidney disease (168). In addition, the clinicians should also be cognizant of the low reliability of HbA1c in this population (187, 188).

### Prevention of Diabetic Kidney Disease

Prior to 2005, primary prevention was the main objective in the management of diabetic kidney disease with optimization of glycemic and hypertension treatment through multifactorial interventions such as lifestyle modification and initiation of pharmacological agents to recommended glycemic and blood pressure targets (189, 190). Metformin has been the first-line therapy recommended by international guidelines for patients with type 2 diabetes, but its use in patients with advanced chronic kidney disease and kidney failure is contraindicated due to the potential and real risk of lactic acidosis (168, 191). It is noteworthy that metformin may have nephroprotective effect *via* activity through glucagon-like peptide-1 (GLP1) receptor, improvement of sodium excretion; and reduction of tubular injury, oxidative stress, inflammation, fibrosis, and apoptosis (192). The practice of avoiding metformin in patients with advanced chronic kidney disease or those maintained on dialysis has been challenged recently with observational studies showing limited association between metformin and the risk of lactic acidosis (193–195). In a recent propensity-matched cohort study of 10,426 patients with type 2 diabetic kidney disease from South Korea, the use of metformin in advanced chronic kidney disease patients, especially those with stage 3B chronic kidney disease, decreased the risk of all-cause mortality and incident kidney failure by over 30%. An increased risk of lactic acidosis was not observed (196). Nevertheless, large randomized controlled trials (RCTs) are required to further evaluate the real-world safety, tolerability, and efficacy of metformin in the treatment of patients with diabetes and advanced chronic kidney disease.

Insulin therapy has been regarded as the default treatment for patients with type 2 diabetes and advanced chronic kidney disease, but weight gain is a relatively common side effect of treatment. Similar to the restricted use of metformin in patients with chronic kidney disease, use of sulfonylureas and meglitinides in patients with advanced chronic kidney disease is often complicated by hypoglycemia, with the use of thiazolidinediones frequently associated with an increased risk of fluid retention and congestive heart failure (197). Although these hypoglycemic agents have consistently shown to improve glycemic control with reduction in HbA1c, none of the traditional agents exhibited noticeable nephroprotective effects in patients with established diabetic kidney disease (168, 198).

### Medical Therapy of Diabetic Kidney Disease

Previously, the only recommended secondary prevention strategy in patients with diabetes and hypertension or albuminuria to retard the progression of kidney disease was angiotensin-converting enzyme inhibitors or angiotensin receptor blockers (ARBs) (191). In 1993, the collaborative study group showed that captopril was associated with a 50% risk reduction of the composite endpoints of death, dialysis, and transplant in patients with insulin-dependent diabetes (181). However, management of diabetes and associated complications have changed markedly since. The Reduction of Endpoints in Non-Insulin-Dependent Diabetes Mellitus with the Angiotensin II Antagonist Losartan (RENAAL) and The Irbesartan Diabetic Nephropathy Trial (IDNT) trials in 2001 showed that ARBs were associated with over 20% risk reduction of doubling of serum creatinine or end-stage kidney disease, independent of the blood pressure-lowering effect (182, 183), but the residual risk remains high between 6 and 8 per 100 patient-years (199).

GLP-1 agonists and dipeptidyl peptidase 4 (DPP4) inhibitors were developed in the mid-2000s. Systematic reviews have shown that GLP-1 agonists improved cardiovascular outcomes with reduction in MACE (HR 0.88, 95% CI 0.82–0.94) and all-cause mortality (0.88, 0.83–0.95) (117), whereas DPP4 studies have failed to show cardiovascular benefits (116, 200). Even though both GLP-1 agonists and DPP4 inhibitors have exhibited minor nephroprotective effects (reduction of microalbuminuria), the impact of hard renal outcomes of delaying or preventing the development of kidney failure remains questionable [The Liraglutide Effect and Action in Diabetes: Evaluation of Cardiovascular Outcome Results (LEADER) and The Cardiovascular and Renal Microvascular Outcome Study With Linagliptin (CARMELINA)] (116, 174).

### Novel Agents

Since 2013, SGLT2 inhibitors have been used in patients with diabetes with early trials establishing the potential cardio- and nephroprotective benefits of these drugs. In 2019, the Canagliflozin and Renal Events in Diabetes and Nephropathy Clinical Evaluation (CREDENCE) trial showed that the exposure to canagliflozin reduced the risk of the primary composite endpoint of end-stage kidney disease, doubling of serum creatinine level, and death from renal or cardiovascular causes by over 30% compared to placebo (180). This landmark study was soon followed by the Dapagliflozin and Prevention of Adverse Outcomes in Chronic Kidney Disease (DAPA-CKD) trial, which showed that the use of dapagliflozin reduced the risk of the composite sustained ≥50% decline in the estimated glomerular filtration rate (eGFR), progression to end-stage kidney disease, or death from renal or cardiovascular causes by almost 40% in patients with eGFR between 25 and 75 ml/min/1.73 m^2^, independent of diabetes status (119, 201). An Australian population prediction model suggested that SGLT2 inhibitors will effectively reduce the incidence of diabetes-related kidney failure in patients with type 2 diabetes by 12%–21% between 2020 and 2040. Nevertheless, the overall incidence rate was projected to trend upwards by 2040 (202). Despite the trial evidence of cardio- and nephroprotective effects of SGLT2 inhibitors, these agents are contraindicated in dialysis patients due to pharmacokinetic dependence of glomerular filtration of glucose for drug efficacy (203).

Several novel agents (aliskiren, bardoxolone, paricalcitol, and sulodexide) were assessed for the potential nephroprotective effect in patients with diabetic kidney disease, but these trials have been disappointing (204–207). Studies of newer agents (atrasentan, baricitinib, ruboxistaurin, pentoxifylline, and finerenone) are promising, with an apparent beneficial effect in reducing microalbuminuria and progression of kidney disease in patients with diabetes (**Table 2**) (118, 122–124, 126, 208, 209). However, further drug efficacy and safety studies are required before these newer agents are considered for clinical practice in patients with diabetic kidney disease.

### Management of Kidney Transplant Recipients With PTDM

The optimal management strategies for the prevention or treatment PTDM remain unknown, with clinical practice guidelines generally informed by non-trial observational data (210). There is currently no trial evidence to support the use of pharmacological therapy to delay or prevent the development of PTDM in kidney transplant recipients, although a small proof-of-concept trial of 50 kidney transplant recipients without diabetes showed that the prescription of basal insulin in the immediate post-transplant period may reduce the incidence of PTDM, possibly by insulin-mediated protection of beta cells (127). Even though immunosuppressive agents have been shown to predispose to the development PTDM, it remains unclear whether the modification of immunosuppressive regimen (e.g., changing from tacrolimus to alternate agents; avoidance, minimization, or split dosing of corticosteroids) can reduce diabetogenic effect or consistently reverse the presence of established PTDM without resulting in a greater risk of other adverse allograft outcomes such as rejection and allograft loss (211–217). The benefit of lifestyle intervention (for weight reduction) or the use of “preventive” pharmacologic treatments including metformin, DPP4-inhibitor, and thiazolidinediones to reduce the risk of progression from a pre-diabetic state to type 2 diabetes in the general population has not been shown for kidney transplant recipients, but these approaches are currently being evaluated in this population group (218–223).

**Figure 3:**
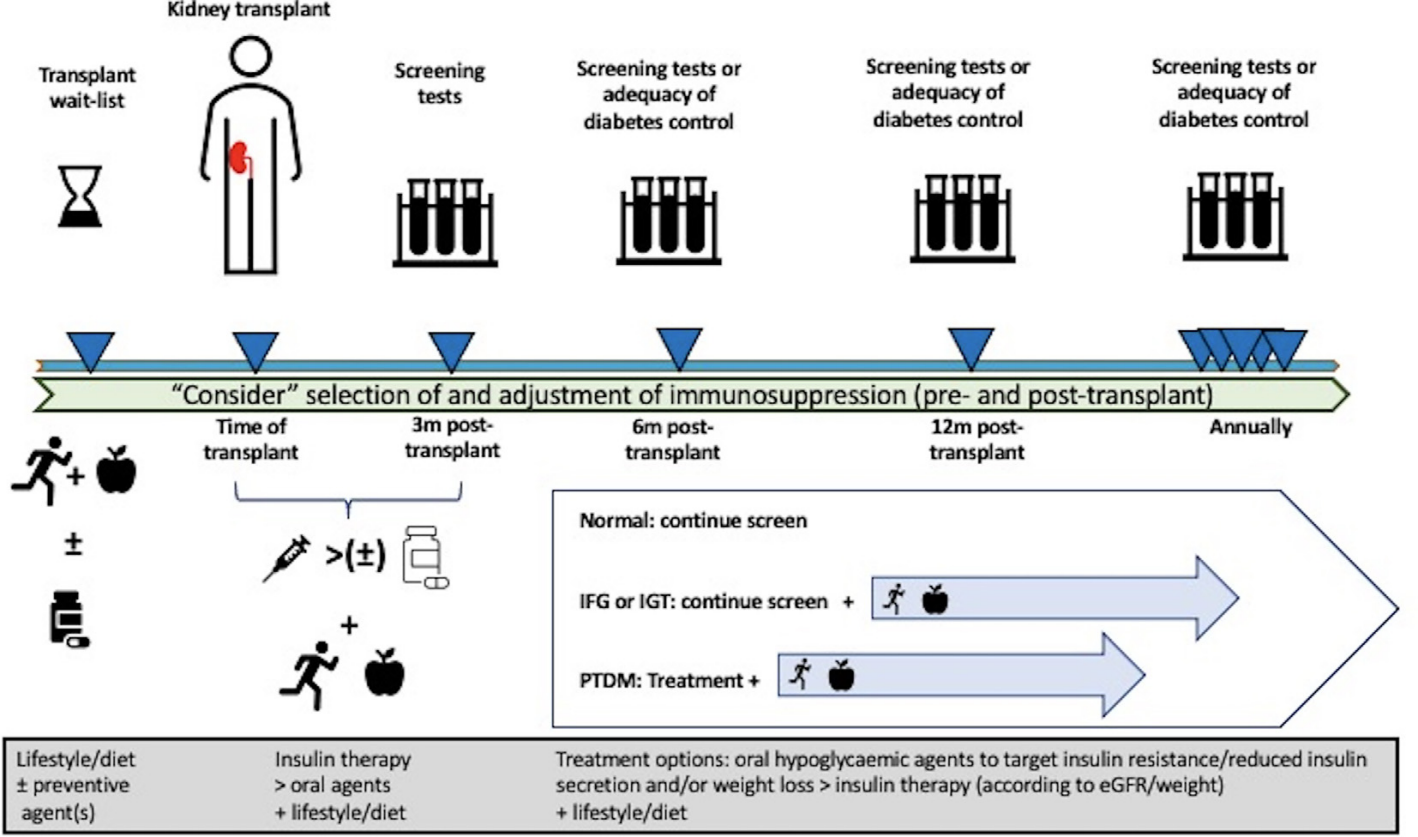
Proposed management algorithm pre- and post-transplantation in the prevention and treatment of post-transplant diabetes mellitus (PTDM) in kidney transplant recipients. Pre-transplant lifestyle intervention and/or preventive pharmacological therapy can be considered to reduce the risk of developing PTDM, but these approaches have not been established for potential kidney transplant candidates. Although the selection and modification of immunosuppression drugs can be considered, no specific immunosuppressive regimen(s) have been shown to prevent the risk of developing PTDM. Following kidney transplantation, consideration of early insulin therapy post-transplant, frequent monitoring for abnormal glucose regulation, lifestyle intervention, and aggressive management of metabolic complications should be undertaken. In kidney transplant recipients with PTDM, the initial treatment with appropriate oral hypoglycemic agents known to improve insulin sensitivity and secretion should be preferred over other hypoglycemic agents and/or insulin therapy.

The approach to the treatment of kidney transplant recipients with PTDM is predictably extrapolated from the general population, with little clinical evidence to support the preferential use of specific oral hypoglycemic agents. The optimal HbA1c in kidney transplant recipients with PTDM is unclear, with the 2009 KDIGO guideline suggesting a target HbA1c of 7.0%–7.5% for recipients with PTDM (ungraded evidence) (66). However, the updated 2020 KDIGO diabetes management guideline recommended individualized HbA1c target ranging from less than 6.5% to less than 8.0% in patients with diabetes and chronic kidney disease not treated with dialysis (level 1C evidence), with this recommendation generalizable to kidney failure patients treated with kidney transplants (168). Given that the predominant underlying mechanisms resulting in PTDM are insulin resistance and/or diminished insulin secretion, the use of oral hypoglycemic agents known to target these pathways may be preferable. A systematic review of seven RCTs, quasi-RCTs, and crossover studies published in 2017 (*n* = 399 kidney transplant recipients with pre-transplant diabetes or PTDM) concluded that the studies were of poor quality and exhibited a high risk of bias, and the findings were unable to inform the superiority of any treatment options (128). Only three studies using DPP4-inhibitor (vildagliptin or sitagliptin vs. placebo or insulin) were undertaken in recipients with PTDM (*n* = 115), with no demonstrable benefit in improving glycemic control, allograft function, or outcome with DPP4-inhibitor (224–227). **Table 4** summarizes the recent literature (case reports, series, and trials) of the use of novel oral hypoglycemic agents in recipients with PTDM. In the limited number of studies of interventions in kidney transplant recipients with PTDM, the efficacy of thiazolidinediones (total of four studies, *n* = 67) (228–230), repaglinide (one study, *n* = 23) (239), GLP1 agonist (four studies in 187 solid organ transplant recipients) (235–238), DPP4-inhibitor (five studies [1 with IGT only], *n* = 110) (224, 225, 231–233), and SGLT2 inhibitor (seven studies, *n* = 92) (227, 240–246) appears to be limited, confounded by the presence of a small number of RCTs and recipients and the difficulty in differentiating the response to intervention of recipients with pre-transplant diabetes compared to those with PTDM. Nevertheless, the limited data do give insight into the safety and tolerability of these novel oral hypoglycemic agents, and the observed lack of impact on the therapeutic drug levels of immunosuppressive agents such as calcineurin-inhibitors is reassuring in the ongoing utility and research of these agents in the treatment of PTDM. A proposed treatment algorithm for the prevention and treatment of PTDM is shown in **Figure 3**.

**Table 4:** Reports of the utilization of novel oral hypoglycemic agents in kidney transplant recipients with post-transplant diabetes mellitus.

Reference (year published)	Drug	Cohort characteristics	Outcomes
**Thiazolidinediones**			
*Luther and Baldwin* (228)	Pioglitazone	Addition of pioglitazone in 10 patients with PTDM treated with insulin or glyburide	Reduction in HBA1c and total daily insulin dose, no impact on tacrolimus level
*Pietruck et al.* (229)	Rosiglitazone	22 patients with PTDM (*n* = 15 tacrolimus, *n* = 7 cyclosporine).	Mean fasting blood glucose reduced in 16 (73%) patients, from 182 ± 17 to 127 ± 7 mg/dl. Edema reported. No impact on tacrolimus and cyclosporine level
*Kurian et al.* (230)	Not specified	19 patients with PTDM	No effect on HBA1c or eGFR
*Werzowa et al.* (231)	Pioglitazone	48 with IGT (3-month double-blind placebo-controlled RCT: 16 to vildagliptin, 16 to pioglitazone, 16 to placebo)	Significant reduction of pioglitazone in HBA1c, fasting and 2-h blood glucose level from baseline to 3-month treatment. Significant reduction in HBA1c compared to placebo
**DPP4-inhibitor**			
*Werzowa et al.* (231)	Vildagliptin	48 with IGT (3-month double-blind placebo-controlled RCT: 16 to vildagliptin, 16 to pioglitazone, 16 to placebo)	Significant reduction of HBA1c and 2-h blood glucose level from baseline to 3 months post-treatment. Significant reduction in HBA1c compared to placebo
*Sanyal et al.* (232)	Linagliptin	21 patients with PTDM (retrospective study) received linagliptin monotherapy for 24 weeks	Significant reduction in fasting and post-prandial blood glucose levels and HBA1c from baseline. No discontinuation or change in tacrolimus level
*Strøm Halden et al.* (225)	Sitagliptin	19 patients with PTDM (crossover study with and without intervention for 4 weeks)	Significantly increased insulin secretion and reduced fasting and postprandial plasma glucose levels. No adverse events and good tolerability
*Boerner et al.* (233)	Sitagliptin	22 patients with PTDM treated with sitagliptin alone	Mean follow-up of 33 months, 17 (77%) remained on sitagliptin. Significant improvement in HBA1c and no effect on calcineurin-inhibitor level or eGFR
*Haidinger et al.* (224, 227)	Vildagliptin	32 patients with PTDM (double-blind placebo-controlled RCT; 16 per group)	Significant reduction in HBA1c and 2-h blood glucose level compared to placebo. Safe and well tolerated
**GLP-1 agonists**			
*Pinelli et al.* (234)	Liraglutide	5 patients (2 with prediabetes and 4 maintained on chronic steroids)	Reduction in blood glucose and weight, no effect on tacrolimus level. No serious adverse events
*Singh et al.* (235)	Dulaglutide	63 solid organ transplant recipients (81% kidney transplant, *n* = 20 with PTDM)	Significantly reduced weight/body mass index and insulin requirements. 6% experienced non-severe hypoglycemic event (at 24-months). No impact on tacrolimus or cyclosporine level
*Thangavelu et al.* (236)	Any of Exenatide, Liraglutide, Dulaglutide, or Semaglutide	19 solid organ transplant recipients (*n* = 7 kidney). Proportion PTDM not specified	Significantly Reduction in weight and HBA1c. Well tolerated with no impact on tacrolimus level or allograft function
*Singh et al.* (237)	Dulaglutide and Liraglutide	88 SOT recipients (*n* = 63 Dulaglutide [81% kidney transplant] and *n* = 25 Liraglutide [84% kidney transplant]). Proportion PTDM not specified	I Improved glycemic control and reduced weight. 15% increased in eGFR with dulaglutide after 24 months. Dulaglutide—6% non-severe hypoglycemia and 3% diarrhea; liraglutide—24% non-severe hypoglycemia and 12% diarrhea
*Kukla et al.* (238)	Liraglutide, Exenatide or Dulaglutide	11/17 patients with PTDM (14/17 kidney-only transplants). 15/17 as add-on therapy	5 (29%) discontinued Significant reduction in insulin dose and non-significant reduction in weight. 5 (29%) discontinued (4 adverse events, 1 lack of efficacy). Well tolerated and no impact on tacrolimus level
**Meglitinides**			
*Türk et al.* (239)	Repaglinide	23 patients with PTDM, with 6 months of follow-up	14 (61%) responded with significant improvement of blood glucose and HbA1c of <7% without the need for additional anti-diabetic agents. No impact on calcineurin-inhibitor level
**SGLT2 inhibitors**			
*Rajasekeran et al.* (240)	Canagliflozin	8/10 of kidney and SPK transplant recipients with PTDM. Mean eGFR 60–78 ml/min/1.73 m^2^	Non-significant reduction in HBA1c, blood pressure and weight. No significant change in eGFR and drug was well tolerated
*Kwon and Kong* (241) *(abstract)*	Dapagliflozin	25 kidney transplant recipients (*n* = 7 with PTDM). 16 (64%) concurrent insulin and/or oral hypoglycemic agents	Reduction in dose of insulin, body weight, and HBA1c. 6 (24%) discontinued drug, 10 (42%) reduced number or dose of anti-hypertensive agents
*Shah et al.* (242)	Canagliflozin	25 with pre-transplant diabetes (*n* = 20) and PTDM (*n* = 5), all with CrCl >60 ml/min	Introduction of canagliflozin reduced the total doses of insulin/other hypoglycemic agents. Reductions in weight, blood pressure, and HBA1c were observed. Well tolerated with no increase in the incidence of infections
*Schwaiger et al.* (243)	Empagliflozin	14 patients with PTDM with eGFR ≥30 ml/min/1.73 m^2^, insulin therapy replaced with empagliflozin	Glucose control inferior to prior exogenous insulin therapy, with 7 (50%) restarted insulin. Reduced oral glucose insulin sensitivity and beta-cell glucose sensitivity. Reduced eGFR with total body volume contraction (and reduced body weight). Safe and well tolerated
*Strøm Halden et al.* (244)	Empagliflozin	44 patients with PTDM (double-blind RCT) with eGFR of >30 ml/min/1.73 m^2^, received 10 mg empagliflozin (*n* = 22) or placebo (*n* = 22). 70% pre-existing glucose-lowering therapies	Significant reduction in HBA1c and body weight with empagliflozin compared to placebo. Adverse events, calcineurin-inhibitor drug levels and eGFR similar
*Mahling et al.* (245)	Empagliflozin	10 patients with PTDM, with empagliflozin as add-on therapy (median eGFR 57 ml/min/1.73 m^2^)	Minor reduction in HBA1c (0.2%), no adverse events and well tolerated
*Attallah and Yassine* (246)	Empagliflozin	8 patients (*n* = 4 with pre-transplant diabetes and *n* = 4 with PTDM). Average creatinine pre-treatment 89 μmol/L	2 of 8 patients developed urinary tract infections. Of the 4 patients with PTDM, reductions in HBA1c and urine proteinuria were observed

Table showing the types, cohort characteristics, and outcomes of novel hypoglycemic agents in the treatment of PTDM.

DPP4-inhibitor, dipeptidyl peptidase-4 inhibitor; GLP-1, glucagon-like peptide 1; SGLT2, sodium-glucose transport protein 2; PTDM, post-transplant diabetes mellitus; HBA1c, glycated hemoglobin; eGFR, estimated glomerular filtration rate; SPK, simultaneous pancreas kidney; CrCl, creatinine clearance; RCT, randomized controlled trial; IGT, impaired glucose tolerance.

## Future Directions

Given the recent trial evidence on the efficacy of novel oral hypoglycemic agents in the treatment of type 2 diabetes in the general population, there is a considerable knowledge gap regarding the safety, tolerability, and efficacy of these newer agents in patients with advanced chronic kidney disease, including those maintained on dialysis or have received kidney transplants. A global collaborative effort composed of epidemiologists, clinicians, trialists, and basic science researchers is required to collect retrospective and prospective data reporting on the use of these and other novel agents in patients with kidney failure, in order to enhance our understanding of the similarities and differences in the pathogenesis of diabetes and related complications in those with kidney failure, identify and validate potential predictive biomarkers to guide evidence-based treatments, and coordinate adequately powered clinical trials to address the knowledge deficiency in the optimal treatment of patients with kidney failure and type 2 diabetes and those with PTDM.

## Conclusion

Our understanding of the incidence and prevalence of type 2 diabetes and related complications among patients with treated kidney failure has significantly advanced with the availability and analysis of big data repositories that showed that diabetic nephropathy has become one of the dominant causes of treated kidney failure worldwide. Data from multiple major linkage projects have shown that treated kidney failure patients with type 2 diabetes have a survival disadvantage compared to those without diabetes, but the magnitude of these risks remains inconsistent. Even though important progress has been made in the understanding of the mechanistic insights into the pathogenesis and ensuing novel treatments to impede kidney disease progression and reduce the burden of vascular complications attributed to diabetes in the last decade, a similar understanding of these novel treatment strategies in patients with diabetes and kidney failure maintained on dialysis or having received kidney transplants remains an elusive goal. Future global collaborative efforts are urgently required to accurately map the disease incidence and prevalence, collating precise age-, gender-, and race-specific data to inform the risk of diabetes-related complications in patients with kidney failure and to provide evidence-based recommendations in the optimal treatment strategies for these patients, including those who have developed PTDM. These data will then inform the development of a global action plan to counteract diabetic kidney disease and provide meaningful knowledge to improve outcomes for the thousands of people with treated kidney failure and diabetes.

## Author Contributions

All authors listed have made a substantial, direct, and intellectual contribution to the work and approved it for publication.

## Conflict of Interest

The authors declare that the research was conducted in the absence of any commercial or financial relationships that could be construed as a potential conflict of interest.

## Publisher’s Note

All claims expressed in this article are solely those of the authors and do not necessarily represent those of their affiliated organizations, or those of the publisher, the editors and the reviewers. Any product that may be evaluated in this article, or claim that may be made by its manufacturer, is not guaranteed or endorsed by the publisher.
